# “Real‐world” performance of the Confirm Rx™ SharpSense AF detection algorithm: UK Confirm Rx study

**DOI:** 10.1002/joa3.13124

**Published:** 2024-09-03

**Authors:** Andre Briosa e Gala, Michael T. B. Pope, Milena Leo, Alexander J. Sharp, Abhirup Banerjee, Duncan Field, Honey Thomas, Richard Balasubramaniam, Ross Hunter, Roy S. Gardner, David Wilson, Mark M. Gallagher, Julian Ormerod, John Paisey, Nick Curzen, Timothy R. Betts

**Affiliations:** ^1^ Department of Cardiology Oxford University Hospitals NHS Foundation Trust Oxford UK; ^2^ Faculty of Medicine University of Southampton Southampton UK; ^3^ Department of Cardiology University Hospitals Southampton Southampton UK; ^4^ Department of Engineering Science Institute of Biomedical Engineering University of Oxford Oxford UK; ^5^ Department of Cardiology East Suffolk and North Essex NHS Foundation Trust Colchester UK; ^6^ Department of Cardiology Northumbria Healthcare NHS Foundation Trust Wansbeck UK; ^7^ Department of Cardiology University Hospitals Dorset NHS Foundation Trust Bournemouth UK; ^8^ Department of Cardiology Barts Health NHS Trust London UK; ^9^ Scottish National Advanced Heart Failure Service Golden Jubilee National Hospital Glasgow UK; ^10^ Department of Cardiology Worcestershire Royal Hospital Worcester UK; ^11^ Department of Cardiology St George's University Hospitals NHS Foundation Trust London UK; ^12^ Department of Cardiology Milton Keynes University Hospital Milton Keynes UK

**Keywords:** ambulatory monitoring, arrhythmia monitoring, atrial fibrillation, implantable cardiac monitor, implantable loop recorders

## Abstract

**Introduction:**

The novel Confirm Rx™ implantable cardiac monitor (ICM) with SharpSense™ technology incorporates a new P‐wave discriminator designed to improve AF detection. This study aimed to evaluate the diagnostic performance of the Confirm Rx™ ICM in detecting AF episodes of varying durations.

**Methods:**

We conducted a multicenter retrospective analysis of consecutive patients implanted with a Confirm Rx™ ICM (v1.2) across nine UK hospitals, all with documented AF lasting at least 6 min. Electrocardiograms (ECGs) were manually adjudicated by cardiologists. To account for intra‐ and inter‐reviewer variability, a random sample of 10% of ECGs underwent additional review. Disagreements were resolved by a third reviewer. Diagnostic performance was determined by calculating the gross and patient‐averaged positive predictive value (PPV) for AF episodes of different duration. The source of false positive (FP) detection was also categorized.

**Results:**

Overall, 16,230 individual ECGs from 232 patients were included. The median AF episode duration was 14 min. R‐wave amplitude remained stable during follow‐up (0.52 ± 0.27 mV [initial] vs. 0.54 ± 0.29 mV [end of follow‐up], *p* = .10). The gross and patient‐averaged PPV were 75.0% and 67.0%, respectively. Diagnostic performance (gross) increased with progressively longer AF episodes: 88.0% for ≥1 h, 97.3% for 6 h, and 100% for 24 h. The main source of FP during tachycardia was T‐wave oversensing (54.2%), while in non‐tachycardic episodes it was predominantly ectopy (71.2%). The AF burden precision was excellent (93.3%).

**Conclusion:**

The Confirm Rx™ ICM diagnostic performance was modest for all AF episodes (75%), with accuracy increasing for longer AF episodes.

## Graphical abstract

Analysis of 16,230 ECGs from 232 patients demonstrated that the Confirm Rx™ ICM had a gross positive predictive value of 75%, increasing to 100% for atrial fibrillation (AF) episodes over 24 h. The trend of improving AF detection accuracy with longer episodes was consistent across all implant indications.

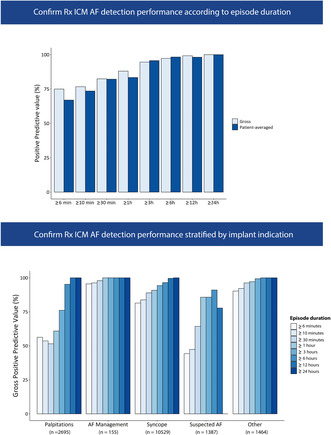

## 1 INTRODUCTION

Atrial fibrillation (AF) diagnosis relies on electrocardiographic (ECG) documentation of irregularly irregular R‐R intervals without any discernible P‐wave.^1^ Twelve‐lead ECGs, Holter monitors, patches and event recorders are routinely employed to detect AF episodes. However, the unpredictable nature of AF results in a relatively low diagnostic yield from intermittent monitoring when compared to prolonged continuous rhythm monitoring with implantable cardiac monitors (ICMs). Recently, there has been a renewed interest in using ICMs for AF diagnosis following cryptogenic strokes^2^, ^3^ and for monitoring AF burden and recurrence after catheter ablation and cardiac surgery.^4^, ^5^, ^6^, ^7^, ^8^

Despite their high sensitivity in detecting AF episodes, ranging between 88 and 96%, theprevious generations of ICMs were limited by the high number of false‐positive (FP) episodes.^9^, ^10^, ^11^ All transmissions require careful adjudication to confirm if episodes are correctly classified as AF, which, given the large volume of data collected, is resource‐intensive and time‐consuming. Moreover, the SARS‐CoV‐2 pandemic has accelerated the adoption of digital solutions, including increased use of remote monitoring of cardiac devices, leading to further strain on already busy device clinics.^12^ Therefore, the diagnostic utility of ICMs relies on accurate and timely detection of clinically significant arrhythmias while minimizing FPs.

Technological advancements have led to minimally invasive ICMs with improved connectivity, remote monitoring capabilities, refined AF algorithms aimed at reducing FP episodes and streamlining workflow. The new Confirm Rx™ ICM (Abbott, Chicago, IL, USA) exemplifies this progress, using wireless telemetry (Bluetooth® and Wi‐Fi/cellular technology) to communicate with the myMerlin™ smartphone app and transmit data to its secure remote monitoring platform (Merlin.net Patient Care Network). A key feature is the SharpSense™ technology, which incorporates a P‐wave discriminator designed to reduce FP detections without compromising AF episode sensitivity. This multicenter, retrospective, *real‐world* study evaluates the diagnostic performance of the Confirm Rx™ ICM with SharpSense™ Technology in detecting AF episodes of varying durations.

## 2 METHODS

The study cohort included consecutive patients from nine UK hospitals implanted with a Confirm Rx™ ICM with SharpSense™ technology (version 1.2) with documented AF episodes lasting at least 6 min and had over 90 days of follow‐up. The ASSERT^13^ study showed increased thromboembolic risk in patients with device‐detected AF ≥6 min, and subsequent studies using ICMs, such as REVEAL‐AF^14^ and LOOP^15^ studies, used the same cut‐off of ≥6 min for clinically significant AF episodes. De‐identified data were extracted from Merlin.net™ remote monitoring database (Abbott; Chicago, IL) and adjudicated by researchers at the Oxford University Hospitals. All participants provided informed consent through the Merlin.net™ Patient Care Network consent form at the time of their ICM implant, authorizing de‐identification, pseudonymization, and data analysis for research purposes. The study was registered and endorsed by Health Regulatory Authority (ID 297175) and received a Research Ethics Committee Review exemption due to its use of previously collected, nonidentifiable information.

### 2.1 Device characteristics and AF algorithm

The Confirm Rx™ ICM with SharpSense™ technology incorporates four new discriminators (three discriminators for bradycardia and pauses and one discriminator for AF) aimed at improving arrhythmia detection accuracy. The original AF algorithm (SJM Confirm), validated against Holter monitors in the DETECT‐AF study, demonstrated high episode sensitivity (94%) and patient sensitivity (100%) but only moderate positive predictive value (64%) for AF lasting at least 2 min duration.^9^ This algorithm relies on monitoring R‐R intervals during a 64‐beat rolling window for three conditions to be met: sudden onset, irregularity, and large variance. A Markov chains model assesses irregularity by comparing measured R‐R intervals to known pattern of AF and non‐AF rhythms. A variance model helps differentiate AF from patterned rhythm, such as bigeminy. The new SharpSense™ algorithm enhances AF detection by adding a P‐wave discriminator, which aims to reduces FP detection by 97% whilst maintaining sensitivity (unpublished data from Abbott).^16^ This discriminator analyses selected P‐waves segments from the preceding 30 s for their morphology and amplitude. AF is only detected if no consistent pattern in the analyzed P‐waves emerges. AF episode parameters including AF duration cut‐off, ECG trigger priority, alert notification, and episode settings are automatically programmed based on the selected reason for monitoring (see Table S1). The nominal R‐wave sensitivity has been increased from 0.150 to 0.125 mV.

Confirm Rx™ ICM uses low‐energy Bluetooth® for secure wireless communication with the myMerlin™ mobile app. This app act as a conduit for episode transmission to the Merlin.net™ Patient Care Network for remote monitoring.

### 2.2 Episode adjudication and statistical analysis

All adjudicated AF episodes had a corresponding 120‐s ECG with heart rate scatterplot, considered representative of the entire episode. A single reviewer (A.B.G, CCDS certified) adjudicated all recordings (episode categorized by the Confirm Rx as AF) and classified them as “True‐AF” or “FP.” To assess for intra‐ and inter‐reviewer variability, a random sample of 10% of stored ECGs was adjudicated by the same reviewer (A.B.G) and a second reviewer (M.T.B.P, CCDS certified). Disagreement was resolved by a third reviewer (T.R.B). The level of agreement between reviewers was calculated using the Cohen's kappa. All reviewers were blinded to ICM indications.

Sensitivity and specificity cannot be calculated from this study, as there is no gold standard, such as a Holter monitor. Therefore, diagnostic performance was evaluated by calculating gross positive predictive value (PPV) and patient‐averaged PPV for AF episodes of different durations (6, 10, 30 min, 1, 3, 6, 12, and 24 h). Gross PPV was computed by dividing all confirmed True‐AF episodes by the total number of AF episodes (True‐AF and FP) detected by the Confirm Rx, assuming each episode is an independent event. Patient‐averaged PPV was calculated by first determining the gross PPV for each patient and then averaging across all patients. Additionally, the investigation of diagnostic performance (PPV) was also extended to predefined groups based on the reason for monitoring, gender, and R‐wave amplitude.

FP episodes were further categorized as due to undersensing, oversensing, noise, atrial/ventricular ectopy, or a combination of the above. AF burden (percentage of time in AF) was computed as the duration of all AF episodes (True‐AF and FP) detected by the Confirm Rx™ divided by the total duration of follow‐up. In contrast, “True‐AF” was calculated by dividing the duration of all “True‐AF” episodes by the total duration of follow‐up. The precision of AF burden then determined by dividing the True AF burden by the total AF burden. We further investigated the performance of AF burden according to ICM implant indication and duration of AF episodes. Statistical analysis was performed using R Statistical Software version 4.0.3. Categorical variables were evaluated for associations using the Fisher's exact test. Nonparametric data from independent groups were compared using the Wilcoxon rank‐sum test, while paired *t*‐tests were used for parametric data from dependent groups.

## 3 RESULTS

Between August 2018 and August 2021, 232 consecutive patients met the inclusion criteria. A total of 16,230 individual recordings were reviewed, demonstrating excellent intra‐ and inter‐observer agreement with Cohen's Kappa values of 0.85 and 0.87, respectively. The patient population was predominantly male (46%), with a median age of 67 years (interquartile range [IQR]: 56–77), and a median duration of follow‐up was 18 months (IQR: 10–22). R‐wave amplitude remained stable with no significant changes from initial implant to end of follow‐up (0.52 ± 0.27 mV vs. 0.54 ± 0.29 mV, *p* = .10). Implant indications are depicted in Table 1. The median episode duration was 14 min (IQR: 9–32), and only 15.1% (2442 episodes) exceeding 1 h in 76 patients. Notably, nearly half of these extended episodes originated from a small subset of patients (2.6%) whose Confirm Rx® ICM were implanted to manage AF. The distribution of age and gender according to implant indication is presented in Table S2.

**TABLE 1:** Baseline demographics and episode characteristics.

Patients, *n*	232
Males, *n* (%)	111 (48%)
Age (years), median (Q1–Q3)	67 (56–77)
Follow‐up (months), median (Q1–Q3)	18 (10–22)
Number of AF episodes	16,230
Number AF episodes per patient, median (Q1–Q3)	10 (3–58)
Implant indication	
AF management	6 (2.6%)
Suspected AF	15 (6.5%)
Palpitations	36 (15.5%)
Syncope	151 (65.1%)
Other	24 (10.3%)
Episode duration (min), median (Q1–Q3)	14 (8.7–31.9)
R wave amplitude (implant), mean ± SD	0.52 ± 0.27 mV
R wave amplitude (follow‐up), mean ± SD	0.54 ± 0.29 mV

Overall, the gross PPV was 75.0%, and patient‐averaged PPV was 67.0% for AF≥6 min (Table 2). Diagnostic performance (gross PPV) increased with progressively longer AF episodes: 88.0% for ≥1 h, 97.3% for ≥6 h, 99.2% ≥ 12 h, and 100% for ≥24 h (graphical abstract). The AF management cohort, despite having the lowest rate of monthly recordings per patient (1.6), exhibited a significantly higher PPV (95.5% True‐AF episodes) compared to other groups (*p* < 0.001; Figure S2). Conversely, the Palpitations (56.3% PPV) and Suspected AF (44.0% PPV) cohorts had the lowest PPV but a substantially higher frequency of monthly recordings (5.1 and 5.8 recordings/patient/month, respectively; Table 3 and Figure S1). Importantly, despite variations in PPV between cohorts, the overall trend of improved performance with increasing episode duration remained consistent across all groups (graphical abstract and Table S3).

**TABLE 2:** Diagnostic performance of the Confirm Rx™ with SharpSense™ Technology for AF episodes of different duration.

Episode duration	Number of AF episodes detected	Number of true AF episodes	Number of patients with true AF	PPV (gross)	PPV (patient‐averaged)
≥6 min	16,230	12,171	188	75.0%	67.0%
≥10 min	10,805	8289	162	76.7%	73.5%
≥30 min	4268	3518	115	82.4%	82.2%
≥1 h	2441	2148	76	88.0%	83.4%
≥3 h	1073	1015	48	94.6%	95.7%
≥ 6 h	622	605	35	97.3%	98.3%
≥12 h	371	368	22	99.2%	98.1%
≥24 h	160	160	14	100%	100%

Abbreviations: AF, atrial fibrillation; PPV, positive predictive value.

**TABLE 3:** Diagnostic performance of the Confirm Rx™ with SharpSense™ Technology as a function of different implant indications.

	Reason for monitoring					Total
	Palpitations	AF management	Syncope	Suspected AF	Other	
Number of episodes of AF detected	2695	155	10,529	1387	1464	16,230
Number of True positive episodes	1516	148	8573	614	1320	12,171
Positive predictive value (gross)	56.3%	95.5%	81.4%	44.3%	90.2%	75.0%
Positive predictive value (patient‐average)	69.3%	81.7%	67.1%	55.2%	65.0%	67.0%

Abbreviation: AF, atrial fibrillation.

**FIGURE 1:**
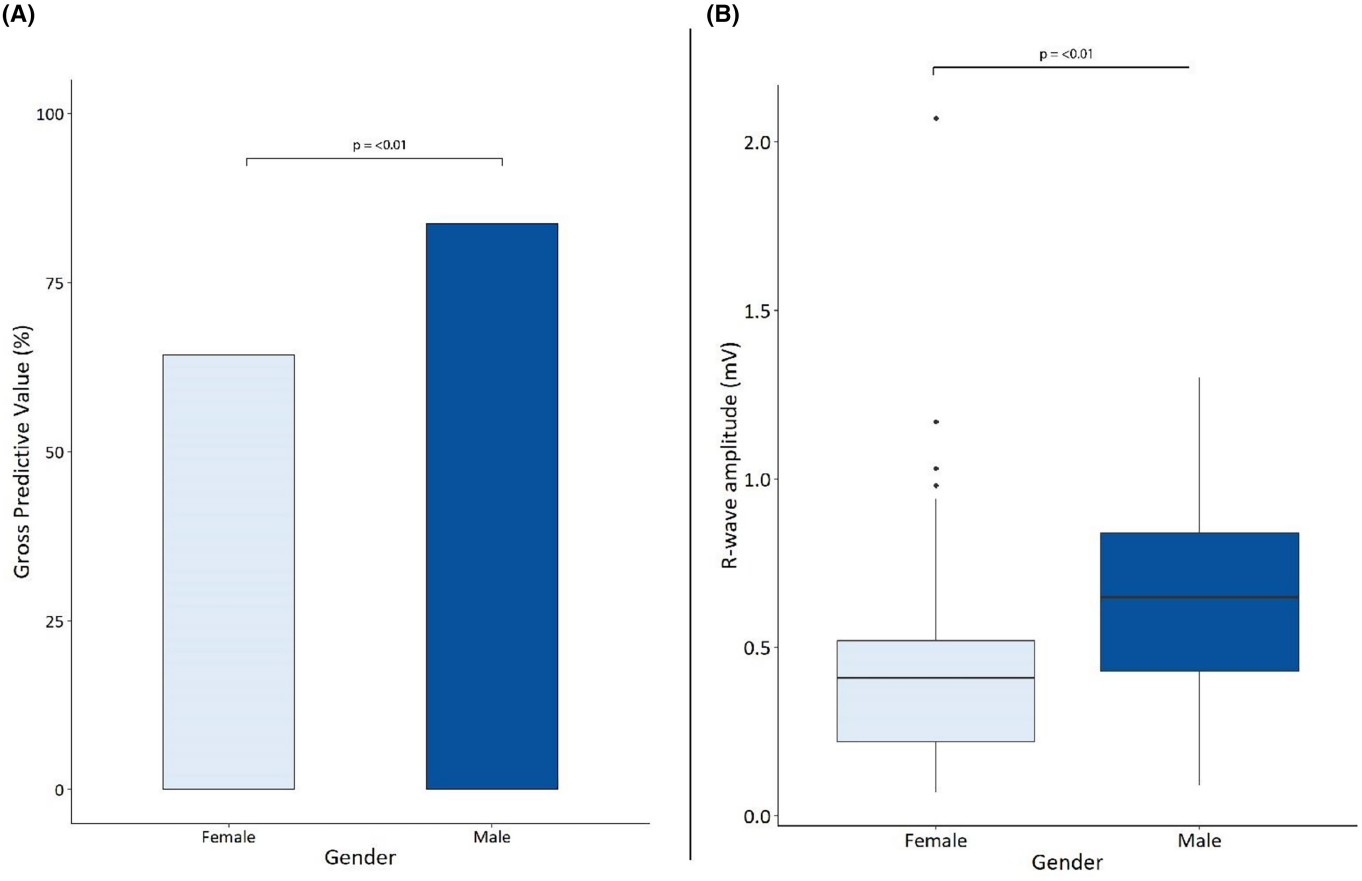
(A) Gender‐based comparison of gross positive predictive value (PPV) for the Confirm Rx. (B) Gender‐based comparison of R‐wave amplitude for the Confirm Rx.

We found a gender‐based difference in both R‐wave amplitude and diagnostic accuracy. Men exhibited a statistically higher median R‐wave amplitude (0.65 mV) compared to women (0.41 mV). This difference correlated with performance, as men had overall higher PPV for AF detection (83.8%) compared to women (64.3%; Figure 1). A noticeable trend of increasing gross PPV was observed with increments of 0.2 mV in R‐wave amplitude (Table S4 and Figure S2). Episodes with R‐wave amplitudes below 0.2 mV had the lowest PPV (53.2%), while those exceeding 1.2 mV achieved a perfect PPV (100%). Further details on PPV and age categories are provided in Table S5 and Figures S3–S5.

**FIGURE 2:**
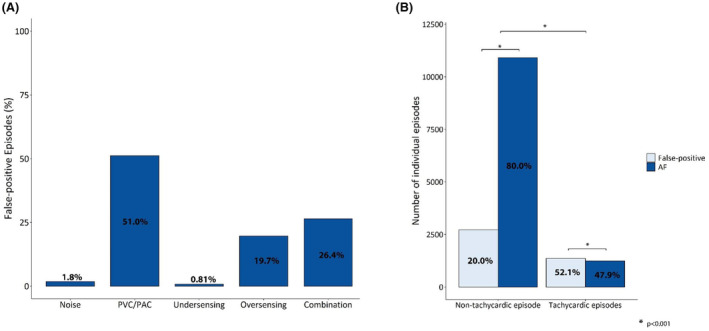
(A) Sources of false‐positive detections for the whole cohort. (B) False‐positive episodes in non‐tachycardic versus tachycardic episodes (defined as mean heart rate ≥100 beats per min).

A quarter (4074) of all episodes classified as AF by the Confirm Rx™ were FP detections in 162 (69.8%) patients. Atrial and ventricular ectopy accounted for approximately half (51.0%) of FP episodes, followed by oversensing (19.7%) and combination of mechanisms (26.4%; Figure 2A). There was statistically significant difference in the rate and distribution of FP episodes according to implant indication (Table S6, Figure S7). Oversensing was responsible for 87.5% of false positives episodes in patients with Confirm Rx for suspected AF. However, for all other implant indications, the predominant source was ectopy, accounting for at least half of the false‐positive episodes observed (Figure S7). Examples can be found in Figures S9–S13.

The Confirm Rx™ performance was lower during tachycardic (mean heart rate >100 bpm) episodes (52.1% vs. 20.0%, *p* < .01; Figure 2B). T‐wave oversensing emerged as the primary culprit for FP detections during tachycardia, particularly in cases of atrial tachycardia/flutter with rapid ventricular response. Interestingly, despite the inclusion of a new P‐wave discriminator in the AF detection algorithm, atrial and ventricular ectopy remained the leading cause (70.8%) of FP events in non‐tachycardic episodes.

**FIGURE 3:**
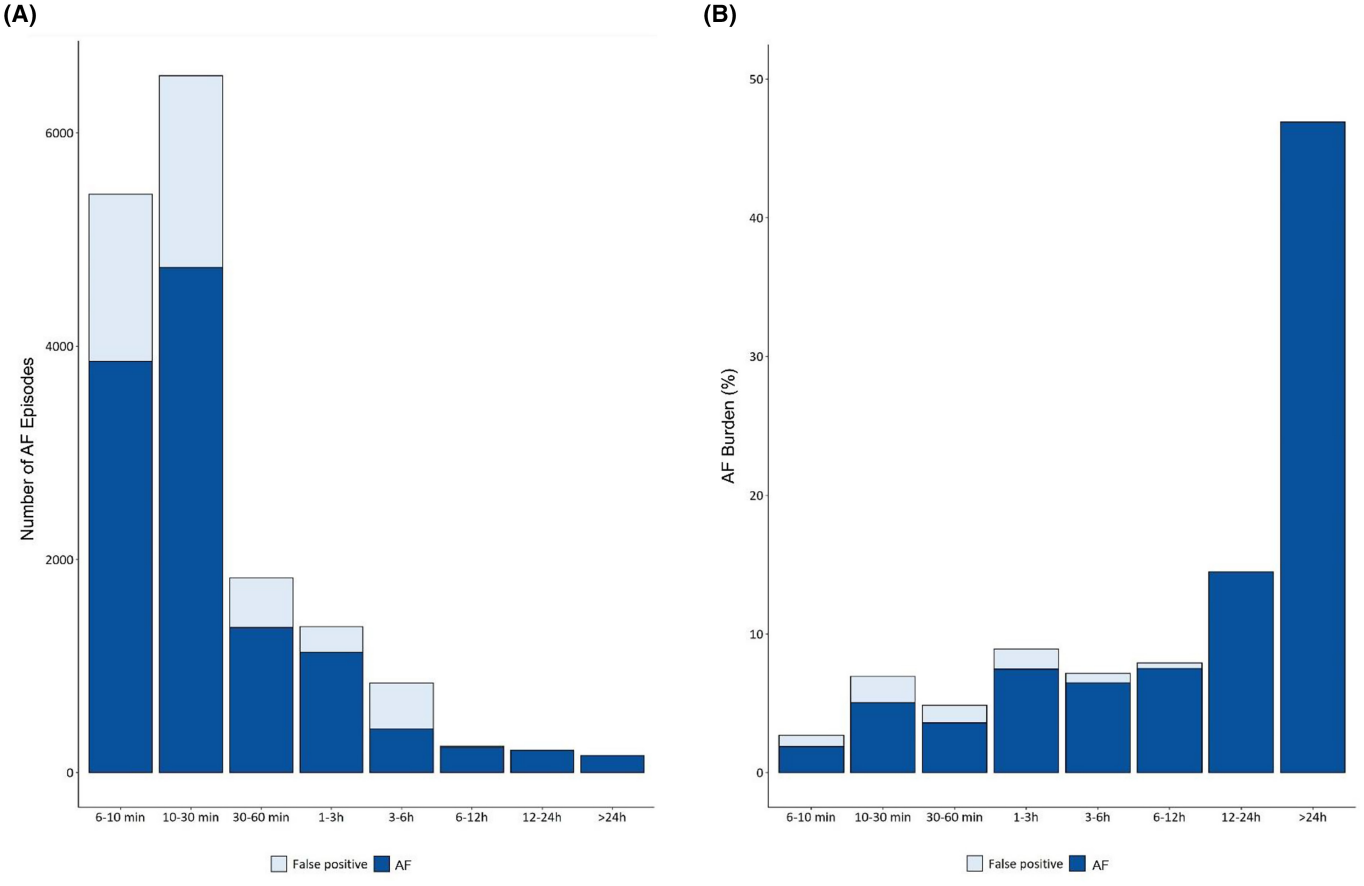
(A) Diagnostic performance of the Confirm Rx™ as a function of episode duration; (B) Contribution of AF episodes of different duration to overall AF burden.

During the follow‐up of 325 patient‐years, a total of 26,137 h of AF episodes were recorded, translating to an overall AF burden of 0.92%. However, a breakdown of these episodes revealed that 24,404 h (or 0.86% of the follow‐up) represented true AF (“True‐AF” burden), with an AF burden precision of 93.3%. The majority of AF episodes (84.5%) were shorter than 1 h, and while approximately a quarter of these were classified as FP, their contribution toward the overall AF burden was minimal. In contrast, AF episodes ≥3 h accounted for 76.4% of time spent in AF, with a very high of “True‐AF” proportion of 98.5% (Figure 3). This explains the observed high performance of estimated AF burden. The patient‐averaged “True‐AF” burden was slightly lower at 82.9%.

## 4 DISCUSSION

The main findings from this “real‐world” study are: (1) the overall diagnostic performance (gross PPV) was 75.0% and was highest with longer AF episodes; (2) the gross PPV was higher in men (83.8%) compared to women (64.3%); (3) despite a new P‐wave discriminator, the dominant mechanism of FP is atrial and ventricular ectopy (51%); and (4) the estimated AF burden for the whole cohort was excellent (93.3%).

Accurate and reliable R‐wave sensing remains critical for any AF detection algorithm to minimize FP events caused by oversensing, undersensing, ectopy, or noise. The miniaturization of the Confirm Rx™ allows for a faster and minimally invasive procedure without compromising signal acquisition. The mean R‐wave amplitude observed in this study 0.54 mV was comparable to the DETECT‐AF^9^ study, that used a larger, prior generation SJM Confirm™ with a different implant technique (0.52 mV). Our findings emphasize the importance of R‐wave amplitude for accurate detection. Episodes with amplitudes below 0.25 mV exhibited a significantly higher rate of FP due to oversensing (35.4% of patients), likely caused by increased sensitivity settings required for such low amplitudes. In contrast, noise and undersensing contributed minimally to FP events (2.6%). Moreover, there was a trend of improved diagnostic accuracy with higher R‐waves (Figure S2).

Consistent with findings from other implantable cardiac monitors (ICMs), we observed a lower median R‐wave amplitude in women compared to men.^17^ The difference in cardiac signal characteristics could be attributed to several factors, including suboptimal positioning of the device, being too superficial and not adjacent to the fascia and muscle layer, influenced by anatomical variations between genders. An additional factor may be signal attenuation caused by the presence of breast tissue. However, this is the first study to demonstrate gender‐based difference in the diagnostic performance of the Confirm Rx™. This may explain our observation that women had higher number of FP due to ectopy and a combination of ectopy and undersensing when compared to men (Figures S6 and S8). There are baseline differences between the studied cohorts albeit not statistically significant. Table S2 shows women had a higher number of implants for syncope but a lower number for suspect AF. This variation could potentially influence the observed FP detection patterns.

The refined AF detection algorithm with SharpSense™ Technology may explain the incremental improvement in the AF diagnostic performance for AF episodes of longer duration. As compared to the DETECT‐AF,^9^ where AF ≥5 min had an overall gross PPV of 62.8% and patient‐averaged PPV of 60.7%, our study with the Confirm Rx™ achieved PPVs of 75% and 66.9%, respectively. Importantly, we observed a gradual reduction in the percentage of FP episodes in episodes of longer duration. AF episodes longer than 1 h had a PPV of 87.0% for all indications, with higher performance in patients with known AF. Despite being widely used, data on the Confirm Rx AF detection algorithm are sparse. Ip et al. randomized 142 patients to either a Reveal‐LINQ ICM or a Confirm Rx.^18^ Twenty patients had 1597 episodes of AF ≥6 min detected by the Confirm Rx, and the patient‐averaged PPV was 38%. The SMART Registry, sponsored by Abbott, is an international prospective observational study (NCT03505801) aiming to recruit 2000 patients to assess the safety of Confirm Rx. However, the diagnostic performance of Confirm Rx is not a predefined outcome measure, and a preliminary analysis reports only the incidence of true arrhythmias.^19^ A recent publication by Gardner et al.^20^ showed that rationalizing ECG adjudication to up to three “key ECGs” reduces the burden of adjudication, but it did not provide any data on the overall performance of Confirm Rx™.

Positive predictive value varies according to the prevalence of AF in the population being studied and AF algorithm settings. The Confirm Rx™ with SharpSense™ Technology AF detections show similar performance to published data on the Reveal LINQ (Medtronic, Minneapolis, MN) using similar methodology. Mittal et al.^21^ reported a gross PPV of 77.5% following the analysis of 13,199 episodes of ≥6 min categorized by the LINQ™ as AF. Incremental gains in performance were also observed in AF ≥1 h and, as the authors highlighted, should guide decisions regarding programming AF duration thresholds to reduce the rate of FP episodes. Nonetheless, Afzal et al.^11^ reported that approximately 99% of ICMs registered in Medtronic's remote monitoring platform have nominal settings rather than being tailored to patients' needs. They reported that over a 4‐week period the rate of FP transmissions from LINQ™ ICMs with nominal setting (AF ≥2 min) ranged from 46% in patients with known AF to 86% in those with cryptogenic stroke.

In a subset of patients, such as those with cryptogenic stroke, AF detection would result in appropriate anticoagulation for secondary stroke prevention, and it is therefore, reasonable that programming is more aggressive (lower AF duration threshold, higher sensitivity) in accepting a larger volume of FP episodes. Following catheter ablation and/or changes in medication, the focus is on burden reduction and correlation of rhythm with symptoms. In research settings, success rates are judged based on freedom of AF of >30 s duration, but in the clinical scenario it may be more appropriate to have alerts set to a longer duration, or based on manual transmissions only.^22^ The Confirm Rx™ AF burden counter includes all episodes, regardless of duration threshold, and cannot be updated following adjudication. This may be considered a limitation; however, we report here that, with SharpSense™ technology, there is a reduction in the rate of FP detections.^23^ As longer episodes, which are far more accurate, dominate in AF burden, there will be a reasonable estimation of the “True‐AF” burden.

P‐wave discriminator analysis is not available during adjudication, which could be potentially valuable to clinicians. In many cases, P‐waves were distinctly present during episodes of atrial ectopy and sinus arrhythmia that were incorrectly labelled as AF, and it is unclear if the P‐wave discriminators cannot detect these small deflections or if P‐waves are incorrectly stacked during the analysis for a consistent P‐ wave pattern. Conversely, other episodes lacked visible P‐wave, yet the heart rate scatterplot clearly showed a regular pattern in keeping with ectopy. The main technical challenge appears to be the low‐amplitude P‐waves in many ECGs. Studies have consistently shown that the ectopy remains the primary source of FP episodes even after the introduction of P‐wave discriminators.^11^, ^18^ As AF detection has historically relied on identifying and analyzing R‐R intervals, ICMs are primarily designed to accurately sense R‐waves and, therefore, the recommended implant location is the left parasternal, 4th intercostal space at a 45‐degree angle. The Confirm Rx™ and LINQ™ have both incorporated P‐wave discriminators to augment their AF detection accuracy, but their implant location does not maximize the P‐wave signal. Indeed, a small study comparing P‐wave visibility in patients with Confirm Rx and Reveal‐LINQ found that in almost 30% of patients the P‐wave was not visible.^24^ Perhaps a less conventional position at the atrial level to improve P‐wave sensing without compromising R‐waves, as seen in some ECG patches, may yield better results and is worth exploring. For example, the Carnation Ambulatory Monitor (CAM) is designed to be a P‐centric ECG patch, and it is applied more medially near the sternum. A small study comparing the CAM to the Zio‐XT patch found that ECGs had higher clarity (100% vs 16%), mostly due to the quality of the P‐wave signal.^25^

The Confirm Rx™ is the first ICM to use Bluetooth® with Wi‐Fi/cellular technology and a smartphone app (myMerlin™) to transmit data to its remote monitoring platform. Initial data from an American registry show a widespread adoption with 97% of 5666 patients registering the app and 92% sending at least one transmission.^26^ Using smartphones instead of traditional bedside monitors is more versatile, allowing transmission from everywhere. The mean time from patient‐activated episodes to Merlin.net availability was only 2.9 min; however, automatic transmission still occurred roughly once a day (mean time 18.5 h).^26^ Improved connectivity opens new management strategies if “real‐time” transmissions and direct patient‐feedback are enabled. For example, a novel concept of “pill‐in‐the‐pocket” oral anticoagulation has been explored in small feasibility studies using ICMs with bedside monitors.^27^, ^28^, ^29^ In REACT.COM,^27^ 59 patients over a mean follow‐up of 1.3 years generated 24,004 transmissions. This workflow is challenging, time‐consuming, expensive, and lacks scalability. A more elegant solution is an ICM which connects with patients' smartphone and alerts them in “real‐time” when AF is detected. Although the current AF episode duration threshold that requires oral anticoagulation is unknown, some studies used AF ≥1 h.^27^, ^28^ In this scenario, there may be a reasonable trade‐off between shortening the time without OAC and the AF detection performance: approximately 1 in 10 patients would restart OAC inappropriately, likely for a few days, until adjudication confirmed a FP episode.

### 4.1 Limitations

First, a “gold‐standard” ambulatory ECG monitor was not available and hence, metrics such as sensitivity and specificity cannot be derived from our dataset. However, DETECT‐AF^9^ reported a high sensitivity for AF episodes making it is unlikely that many AF episodes were missed. PPV is a useful parameter of diagnostic performance and can be used to compare the Confirm Rx™ to other ICMs. Moreover, the limited duration of ambulatory monitoring restricts the analysis of longer AF episodes and the assessment of overall AF burden. Second, we had limited information regarding baseline characteristics, such as body mass index and implant location, which could affect the overall quality of ECG recordings and sensed R‐waves. Third, we considered the first 120 s of the ECG to represent the entire episode when estimating the performance of AF episode detection and AF burden. Finally, this study reflects UK practice where syncope is the commonest indication for an ICM. The PPV for each cohort should be interpreted with caution due to the imbalance in the number of patients.

## 5 CONCLUSION

The diagnostic performance of the AF algorithm in the novel Confirm Rx with SharpSense™ technology was 75%, with the highest accuracy observed in longer AF episodes. Confirm Rx performance was significantly better in men than women, with rates of 83.8% and 64.3%, respectively. R ‐wave parameters were excellent and remained stable during follow‐up. The Confirm Rx™ AF detection demonstrates an improved performance compared to its previous iteration. However, ectopy is still accounts for most FP episodes despite the introduction of P‐wave discriminators.

## FUNDING INFORMATION

None.

## CONFLICT OF INTEREST STATEMENT

ABG, RSG, and TB have received financial support for research from Abbott. MTBP received financial support to attend scientific meetings from Acutus Medical. HT received financial support to attend scientific meetings from Abbott, BSCI, Medtronic, and Boston Scientific. DW has received financial support to attend scientific meetings. AJS, AB, JRP, ML, NC, MGM, RB, JO, and DF– no conflicts in relation to this paper.

## ETHICS APPROVAL

Exempt from REC within UK Health Departments' Research Ethics Services, as this is research limited to the use of previously collected, nonidentifiable information, where patients have given prior consent at the time of implantable cardiac monitor insertion.

## PATIENT CONSENTED

The content of this manuscript adheres to the Wiley's Best Practice Guidelines on Research Integrity and Publishing Ethics.

## IRAS PROJECT NUMBER

297175.

## CLINICAL TRIALS REGISTRATION

Not applicable.

## Supporting information


Data S1.

## Data Availability

Raw data available upon reasonable request.
