# Supplementary material for: “Real‐world” performance of the Confirm Rx™ SharpSense AF detection algorithm: UK Confirm Rx study
Source: J Arrhythm. 2024 Sep 3;40(5):1093–101. doi: 10.1002/joa3.13124 (PMC11474619; doi:10.1002/joa3.13124)
Supplement: Supplementary file 1 — Data S1. [file JOA3-40-1093-s001.docx]

**Supplementary Material**

‘Real-world’ performance of the Confirm Rx™ SharpSense AF detection algorithm: UK Confirm Rx study

This supplementary material has been provided by the authors to give readers additional information about their work.

**Table of contents**

[**Table S1.** Confirm Rx settings according to reason for monitoring automatic parameters (from Confirm Rx™ manual [Abbott]). 2](#_Toc171087386)

[**Table S2.** Age and sex according to implant indication. 3](#_Toc171087387)

[**Figure S1.** Number of recordings per patient month for different implant indications. 4](#_Toc171087388)

[**Table S3.** Diagnostic performance of AF detection algorithm for episodes of different durations and according to implant indication. 5](#_Toc171087389)

[**Table S4.** Summary of patients and episodes based on R-wave amplitude. 6](#_Toc171087390)

[**Figure S2.** Diagnostic Performance of AF Detection Algorithm based on R-Wave Amplitude. 7](#_Toc171087391)

[**Figure S3.** Distribution of age category in the study cohort. 8](#_Toc171087392)

[**Table S5.** Summary of patients and episodes based on age. 9](#_Toc171087393)

[**Figure S4.** R-wave amplitude in different age groups 10](#_Toc171087394)

[**Figure S5.** Diagnostic performance of AF detection algorithm based on age. 11](#_Toc171087395)

[**Table S6.** False-positive detections according to implant indications. 12](#_Toc171087396)

[**Figure S6.** Distribution of types of false-positive detections according to gender. 13](#_Toc171087397)

[**Figure S7.** False-positive detections according to implant indications. 14](#_Toc171087398)

[**Figure S8.** Number of false-positive episodes according to R-wave amplitude and gender. 15](#_Toc171087399)

[**Figure S9.** Example of True-AF episode. 16](#_Toc171087400)

[**Figure S10.** Example of false positive episode due to ventricular ectopy. 17](#_Toc171087401)

[**Figure S11.** Example of false-positive episode due to oversensing and ventricular ectopy. 18](#_Toc171087402)

[**Figure S12**. Example of false-positive episode due to T-wave oversensing. 19](#_Toc171087403)

[**Figure S13** Example of false-positive episode due to undersensing. 20](#_Toc171087404)

# **Table S1.** Confirm Rx settings according to reason for monitoring automatic parameters (from Confirm Rx™ manual [Abbott]).

| **Reason for monitoring** | **AF duration** | **AF** | **Pause** | **Tachycardia** | **Bradycardia** | **Tachycardia sudden onset** |
| --- | --- | --- | --- | --- | --- | --- |
| **Syncope** | 6 minutes | Low | High | High | High | On |
| **Seizures** | 6 minutes | High | High | High | High | On |
| **Palpitations** | 6 minutes | High | Low | High | Low | On |
| **Ventricular Tachycardia** | 10 minutes | Low | Low | High | Low | Off |
| **Suspected AF** | 6 minutes | High | Low | Low | Low | On |
| **Post AF Ablation** | 6 minutes | High | Low | Low | Low | On |
| **AF management** | 6 minutes | High | Low | Low | Low | On |
| **Cryptogenic Stroke** | 2 minutes | High | Low | Low | Low | On |
| **Other** | 2 minutes | High | High | Low | Low | Off |

# **Table S2.** Age and sex according to implant indication.

|  | **Palpitations** | **AF Management** | **Syncope** | **Suspected AF** | **Others** |
| --- | --- | --- | --- | --- | --- |
| **Age** *(years)* | 54 (37-67) | 70 (63-76) | 72 (57-82) | 67 (57-67) | 64 (57-77) |
| **Sex** |  |  |  |  |  |
| **Female** | 3 (50%) | 12 (52.1%) | 22 (64.8%) | 5 (38.4%) | 64 (42.1%) |
| **Male** | 3 (50%) | 11 (47.8%) | 12 (35.2%) | 8 (61.5%) | 78 (54.9%) |

^1^ Krustal-Wallis test; Fisher exact test


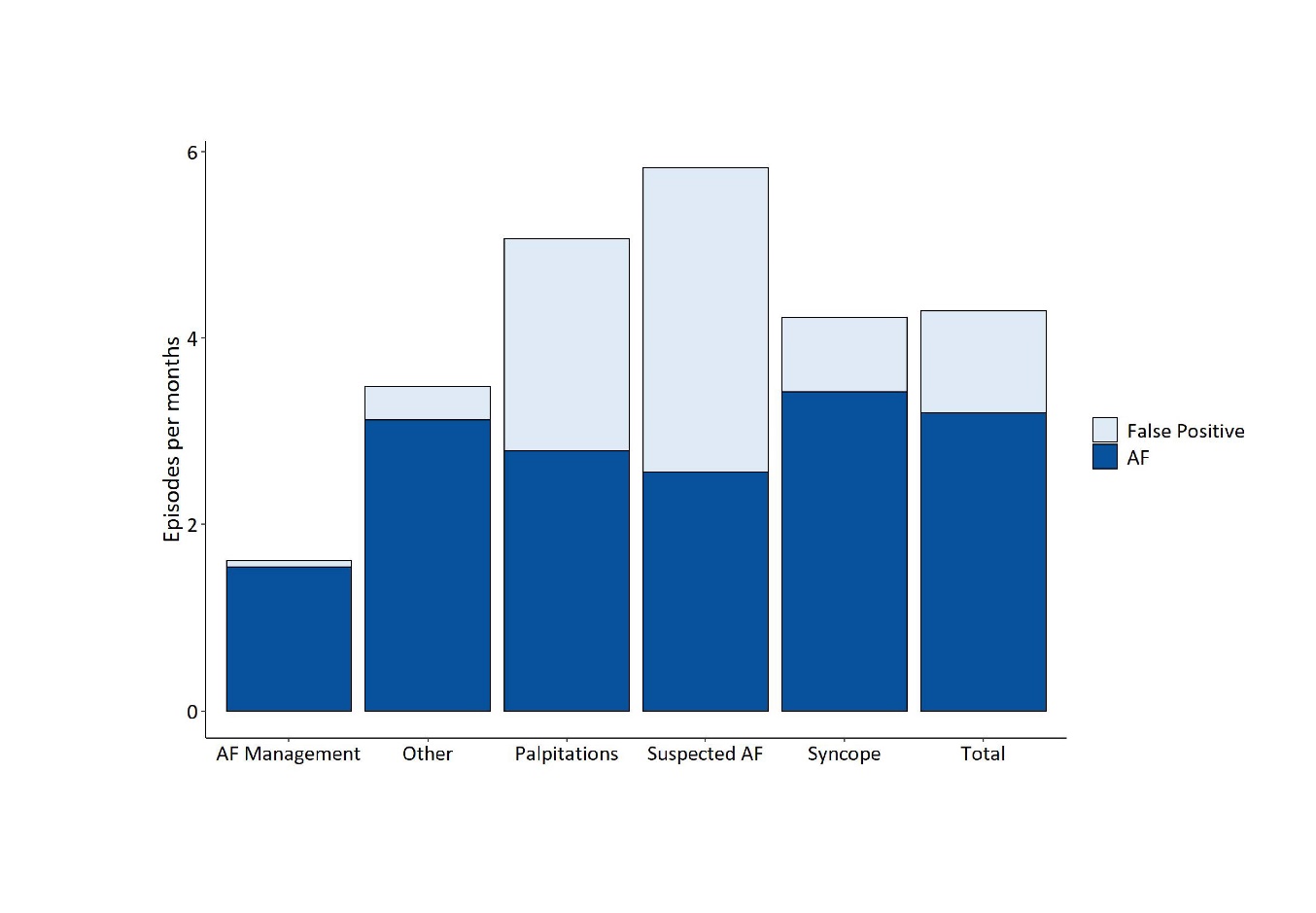


# **Figure S1.** Number of recordings per patient month for different implant indications.

# **Table S3.** Diagnostic performance of AF detection algorithm for episodes of different durations and according to implant indication.

| **Indication** | **Episode duration** | **Number of AF episodes detected** | **Number of True AF episodes** | **PPV** |
| --- | --- | --- | --- | --- |
| **Palpitations** (n=36) | >6 minutes | 2695 | 1,516 | 56.3% |
|  | >10 minutes | 1765 | 946 | 53.6% |
|  | >30 minutes | 656 | 338 | 51.5% |
|  | >1h | 322 | 196 | 60.9% |
|  | >3h | 67 | 51 | 76.1% |
|  | >6h | 21 | 20 | 95.2% |
|  | >12h | 8 | 8 | 100% |
|  | >24h | 6 | 6 | 100% |
|  | >6 minutes | 155 | 148 | 95.5% |
|  | >10 minutes | 131 | 126 | 96.2% |
|  | >30 minutes | 97 | 95 | 97.9% |
| **AF Management** (n=6) | >1h | 73 | 73 | 100% |
|  | >3h | 49 | 49 | 100% |
|  | >6h | 37 | 37 | 100% |
|  | >12h | 24 | 24 | 100% |
|  | >24h | 3 | 3 | 100% |
|  | >6 minutes | 10529 | 8573 | 81.4% |
|  | >10 minutes | 6817 | 5714 | 83.8% |
|  | >30 minutes | 2498 | 2221 | 88.9% |
| **Syncope** (n=151) | >1h | 1417 | 1286 | 90.8% |
|  | >3h | 649 | 612 | 94.3% |
|  | >6h | 404 | 391 | 96.5% |
|  | >12h | 256 | 255 | 99.6% |
|  | > 24h | 133 | 133 | 100% |
|  | >6 minutes | 1387 | 614 | 44.3% |
|  | >10 minutes | 946 | 447 | 47.3% |
|  | >30 minutes | 359 | 231 | 64.3% |
|  | >1h | 161 | 138 | 85.7% |
| **Suspected AF** (n=15) | >3h | 36 | 33 | 91.7% |
|  | >6h | 15 | 13 | 86.7% |
|  | >12h | 9 | 7 | 77.8% |
|  | >24h | - | - | - |
|  | >6 minutes | 1464 | 1320 | 90.2% |
|  | >10 minutes | 1148 | 1156 | 92.1% |
|  | >30 minutes | 658 | 633 | 96.2% |
|  | >1h | 468 | 455 | 97.2% |
| **Other** (n=24) | >3h | 272 | 270 | 99.3% |
|  | >6h | 144 | 144 | 100% |
|  | >12h | 18 | 17 | 94.4% |
|  | >24h | 13 | 13 | 100% |

**AF**: atrial fibrillation; **PPV**: positive predictive value.

| **R-wave categories**  **(mV)** | **Number of patients** | **Number of AF episodes detected** | **Number of True-AF episodes** |
| --- | --- | --- | --- |
| 0-0.19 | 20 | 1952 | 1038 |
| 0.2-0.39 | 49 | 3757 | 2136 |
| 0.4-0.59 | 63 | 4235 | 3589 |
| 0.6-0.79 | 29 | 1631 | 1290 |
| 0.8-0.99 | 19 | 2440 | 2131 |
| 1.0-1.19 | 19 | 498 | 443 |
| 1.2-1.39 | 1 | 162 | 162 |
| 1.4-1.59 | 0 | 0 | 0 |
| 1.6-1.79 | 0 | 0 | 0 |
| 1.8-1.99 | 0 | 0 | 0 |
| >2 | 1 | 2 | 2 |

# **Table S4.** Summary of patients and episodes based on R-wave amplitude.


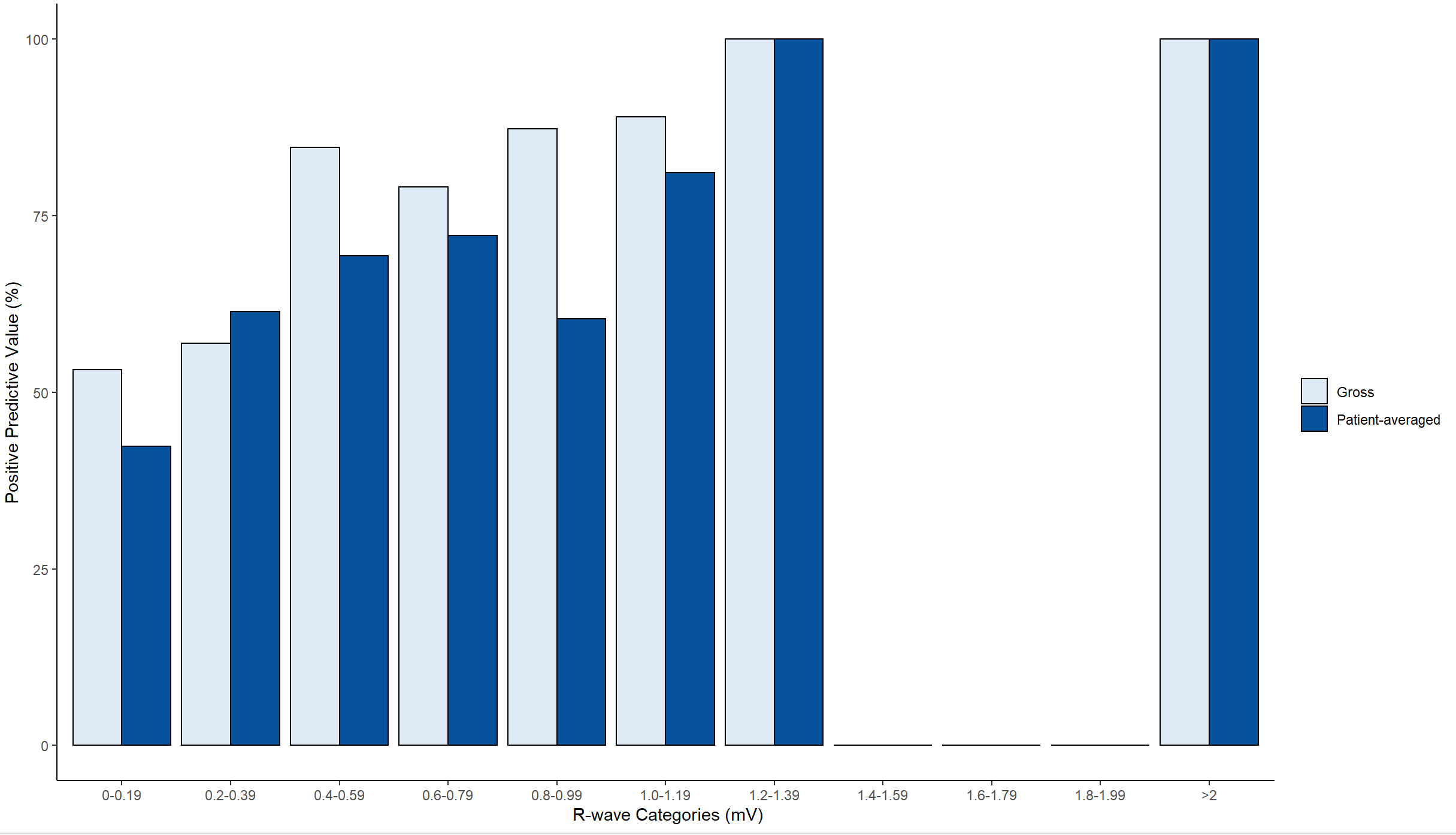


# **Figure S2.** Diagnostic Performance of AF Detection Algorithm based on R-Wave Amplitude.

**
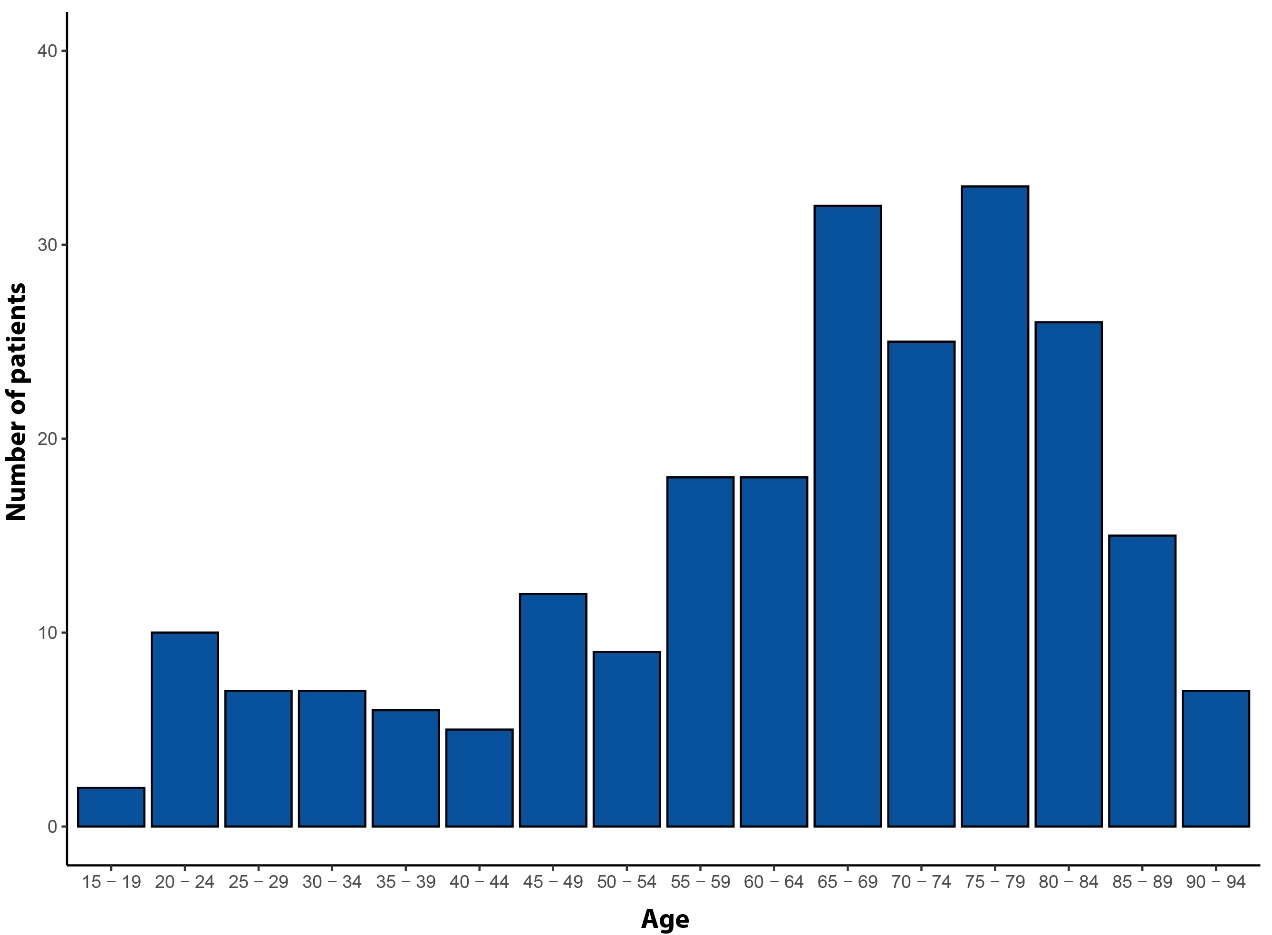
**

# **Figure S3.** Distribution of age category in the study cohort.

# **Table S5.** Summary of patients and episodes based on age.

| **Age Categories**  **(years)** | **Number of patients** | **Number of AF episodes detected** | **Number of True-AF episodes** |
| --- | --- | --- | --- |
| **15 - 19** | 2 | 365 | 333 |
| **20 - 24** | 10 | 768 | 704 |
| **25 - 29** | 7 | 579 | 497 |
| **30 - 34** | 7 | 71 | 52 |
| **35 - 39** | 6 | 458 | 280 |
| **40 - 44** | 5 | 226 | 158 |
| **45 - 49** | 12 | 322 | 142 |
| **50 - 54** | 9 | 359 | 202 |
| **55 - 59** | 18 | 212 | 148 |
| **60 - 64** | 18 | 1073 | 960 |
| **65 - 69** | 32 | 2259 | 1,653 |
| **70 - 74** | 25 | 2280 | 894 |
| **75 - 79** | 33 | 1801 | 1,445 |
| **80 - 84** | 26 | 4364 | 3,780 |
| **85 - 89** | 15 | 963 | 810 |
| **90 - 94** | 7 | 130 | 113 |


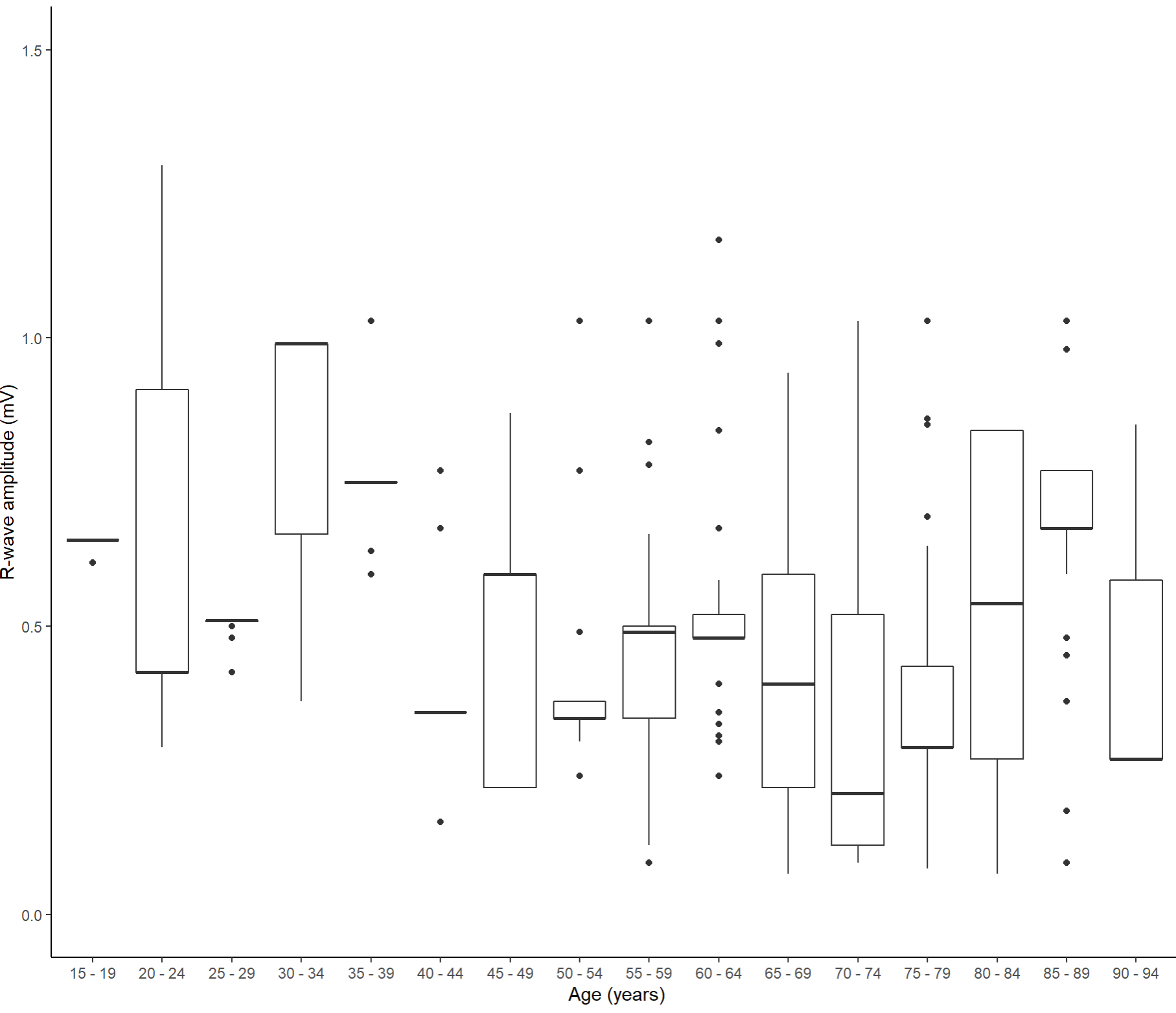


# **Figure S4.** R-wave amplitude in different age groups


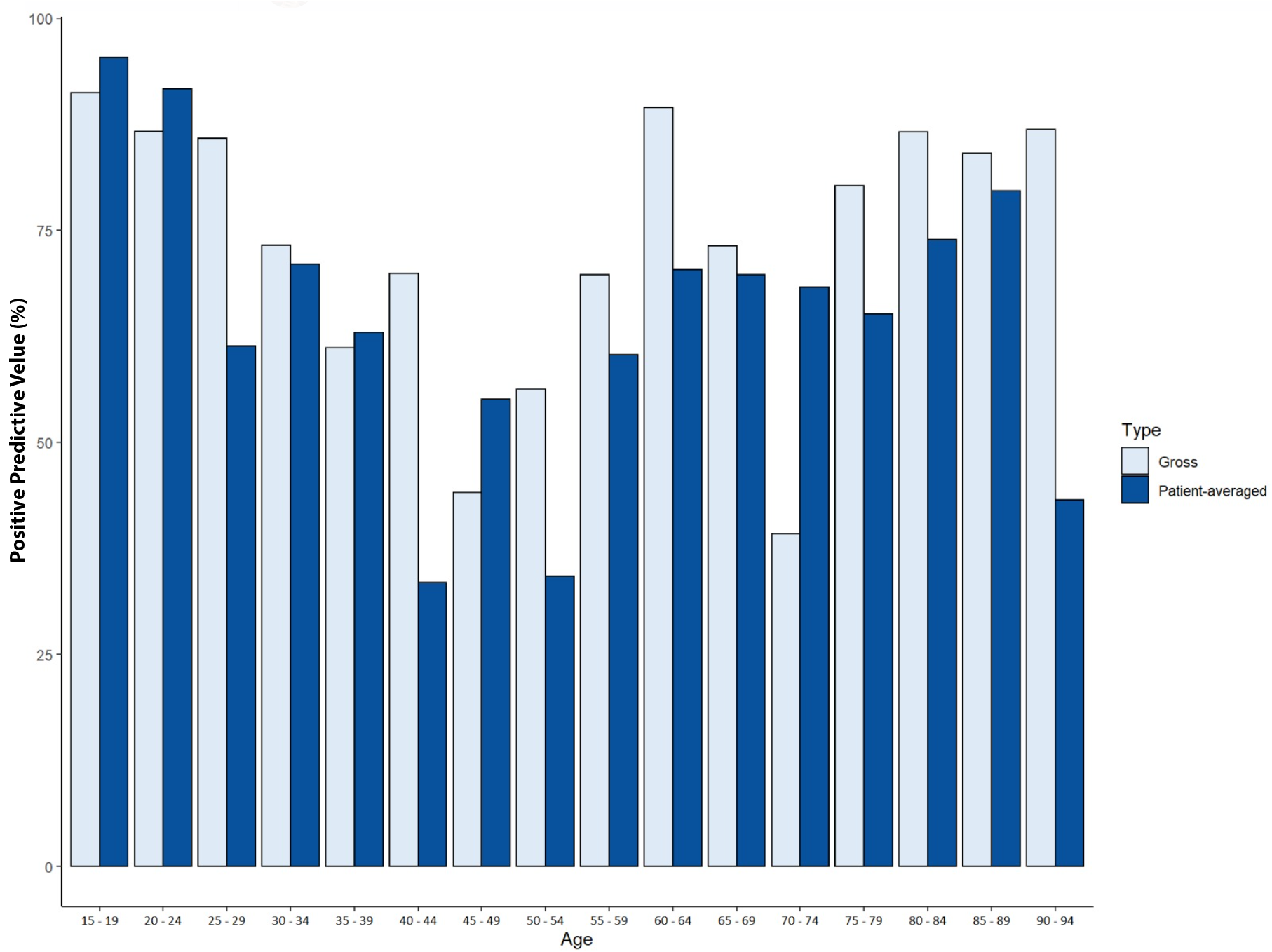


# **Figure S5.** Diagnostic performance of AF detection algorithm based on age.

|  | **Palpitations** | **AF Management** | **Syncope** | **Suspected AF** | **Others** | **p-value^1^** |
| --- | --- | --- | --- | --- | --- | --- |
| **False-positive** | 1187 (44%) | 7 (4.5%) | 1964 (19%) | 774 (56%) | 146 (10%) | <0.001 |
| **True-AF** | 1516 (56%) | 148 (95.5%) | 8573 (81%) | 614 (44%) | 1320 (90)% | <0.01 |

# **Table S6.** False-positive detections according to implant indications.

^1^Chi-square test of independence


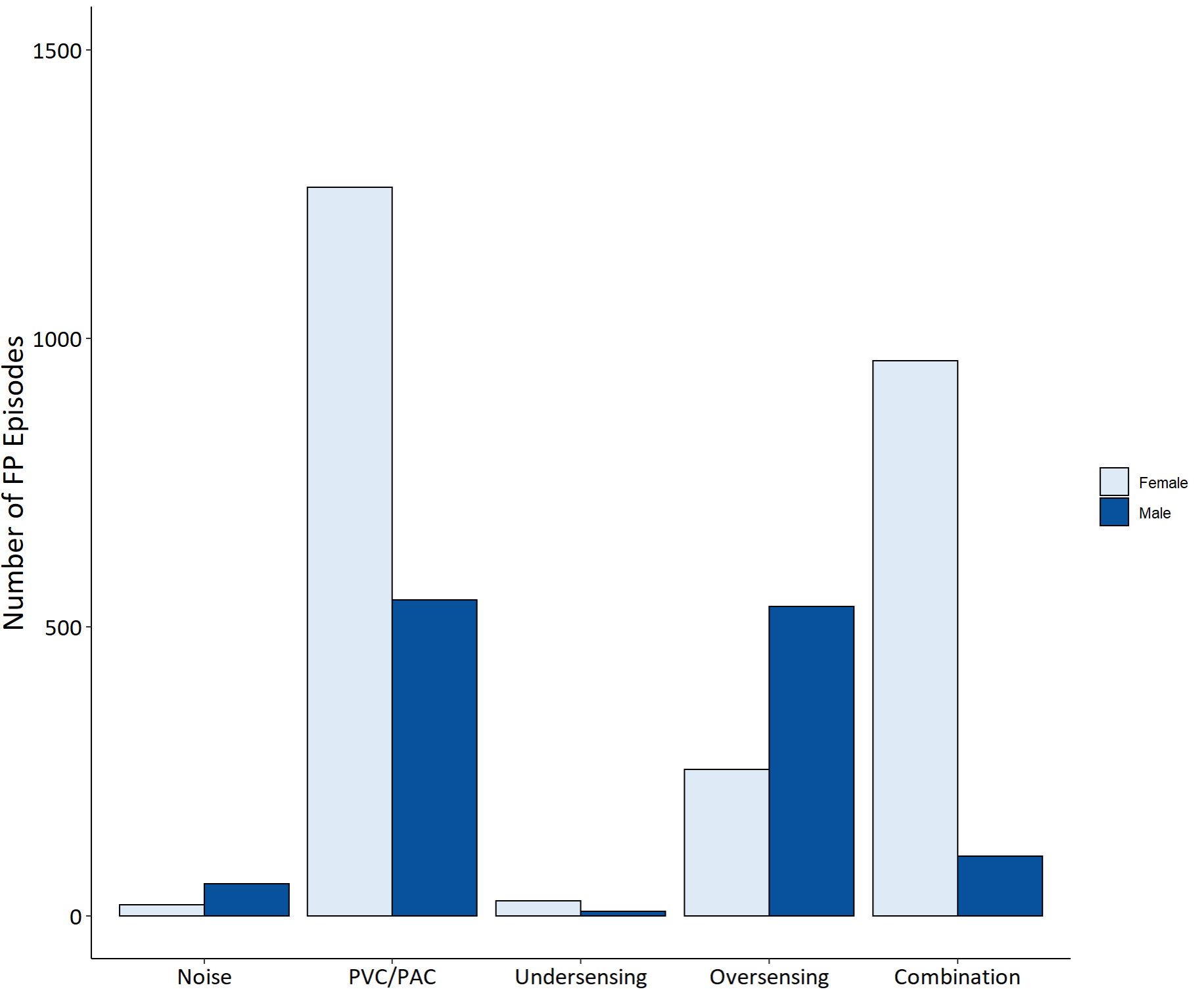


# **Figure S6.** Distribution of types of false-positive detections according to gender.


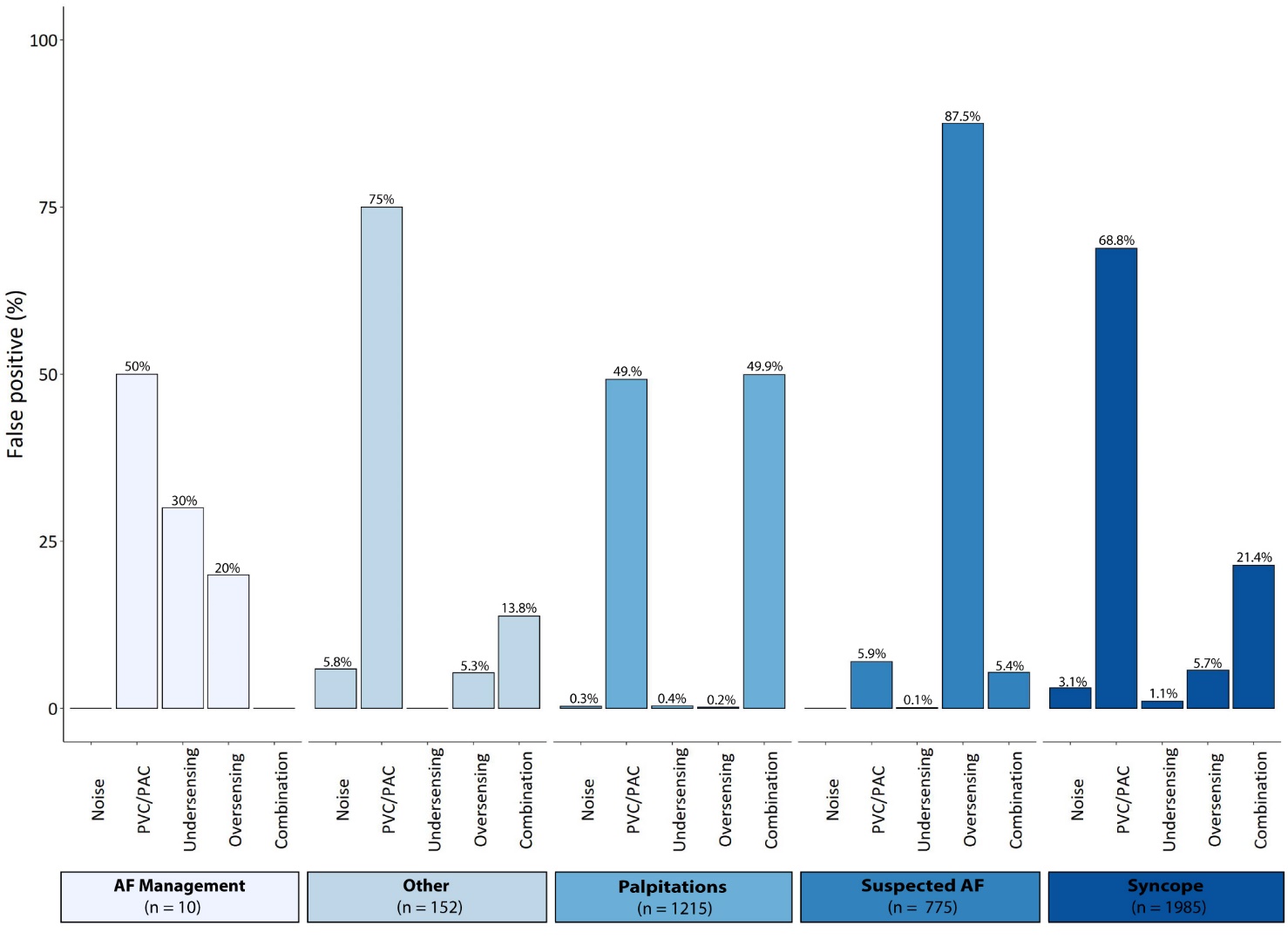


# **Figure S7.** False-positive detections according to implant indications.


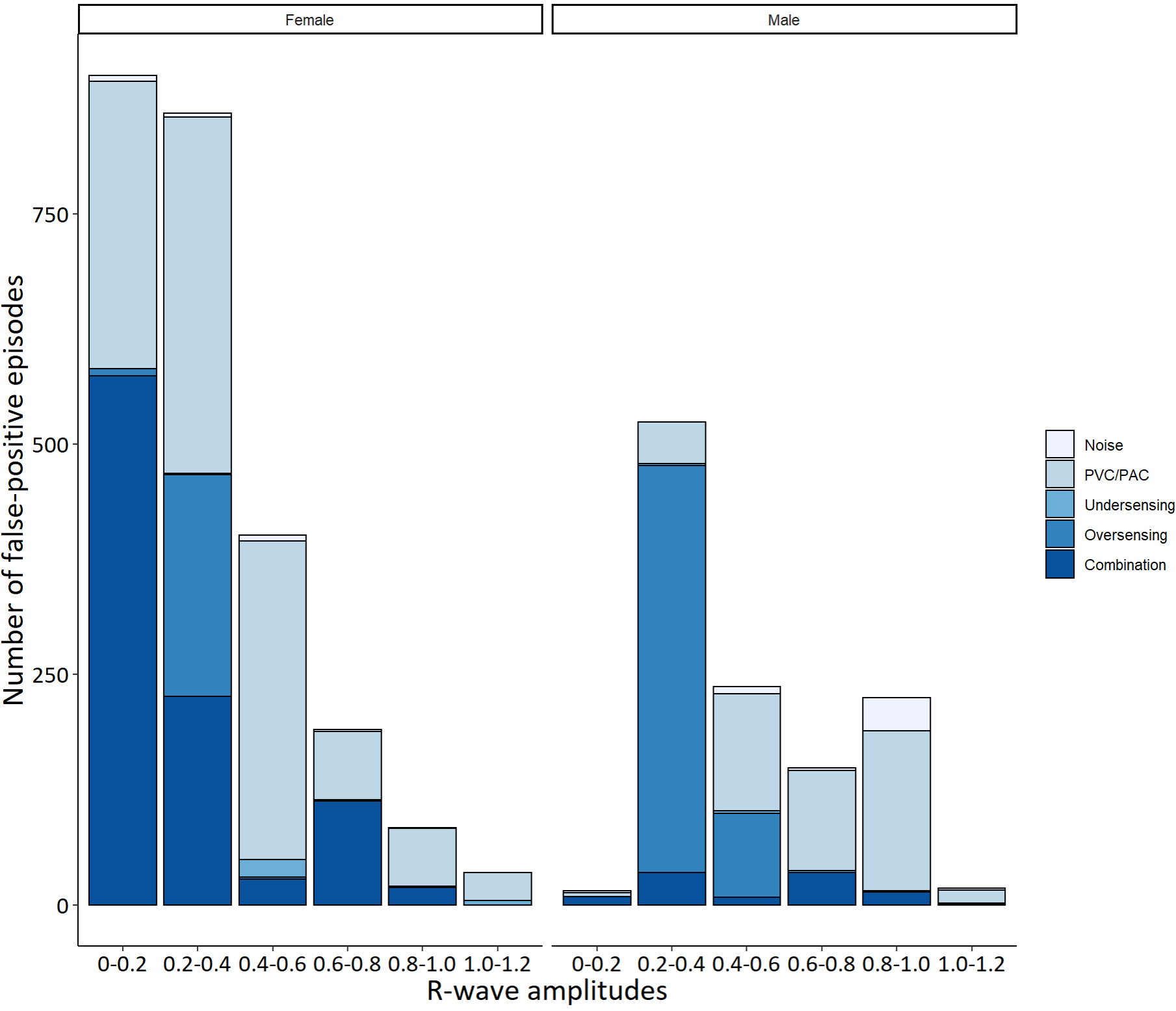


# **Figure S8.** Number of false-positive episodes according to R-wave amplitude and gender.


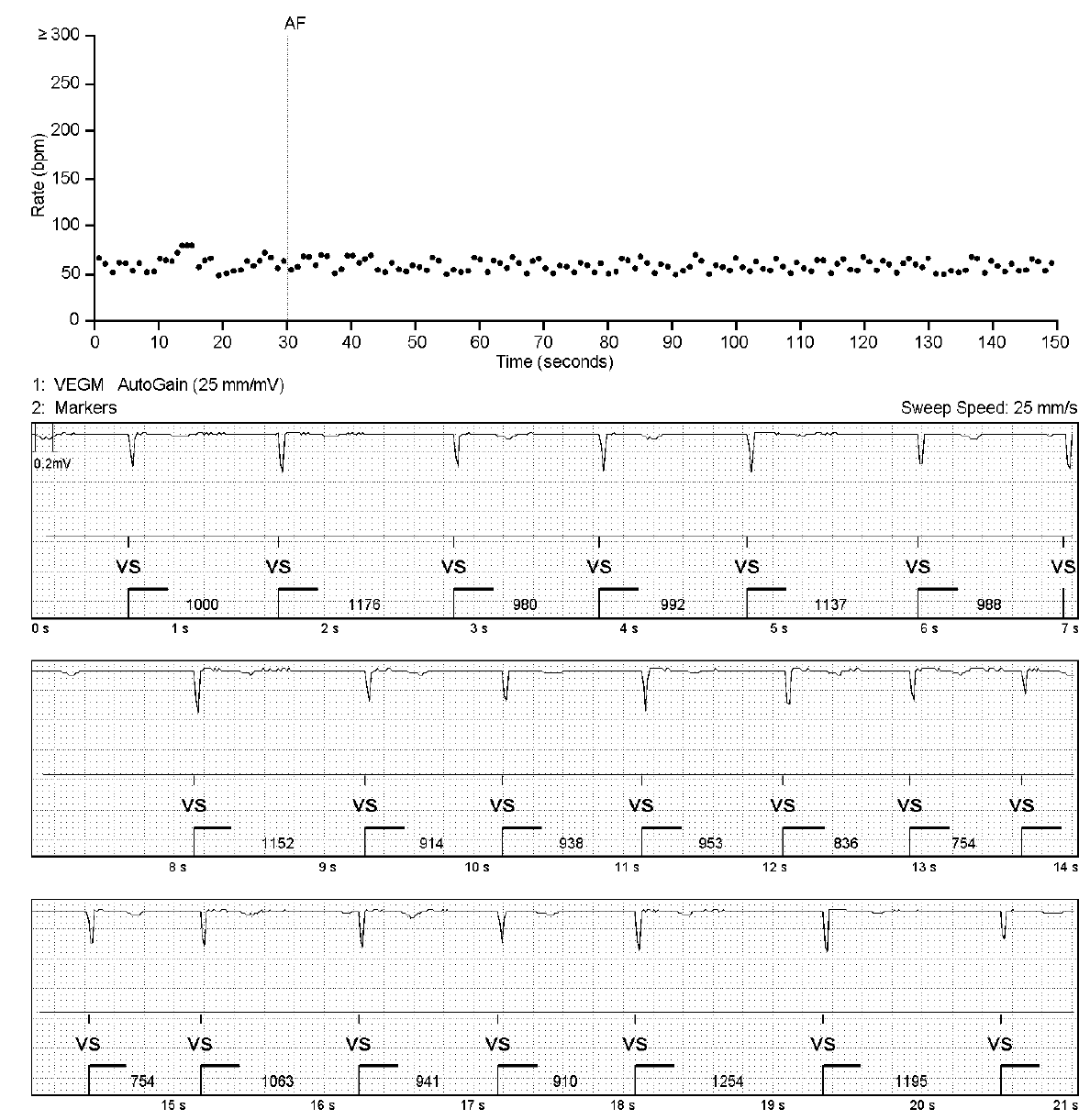


# **Figure S9.** Example of True-AF episode.


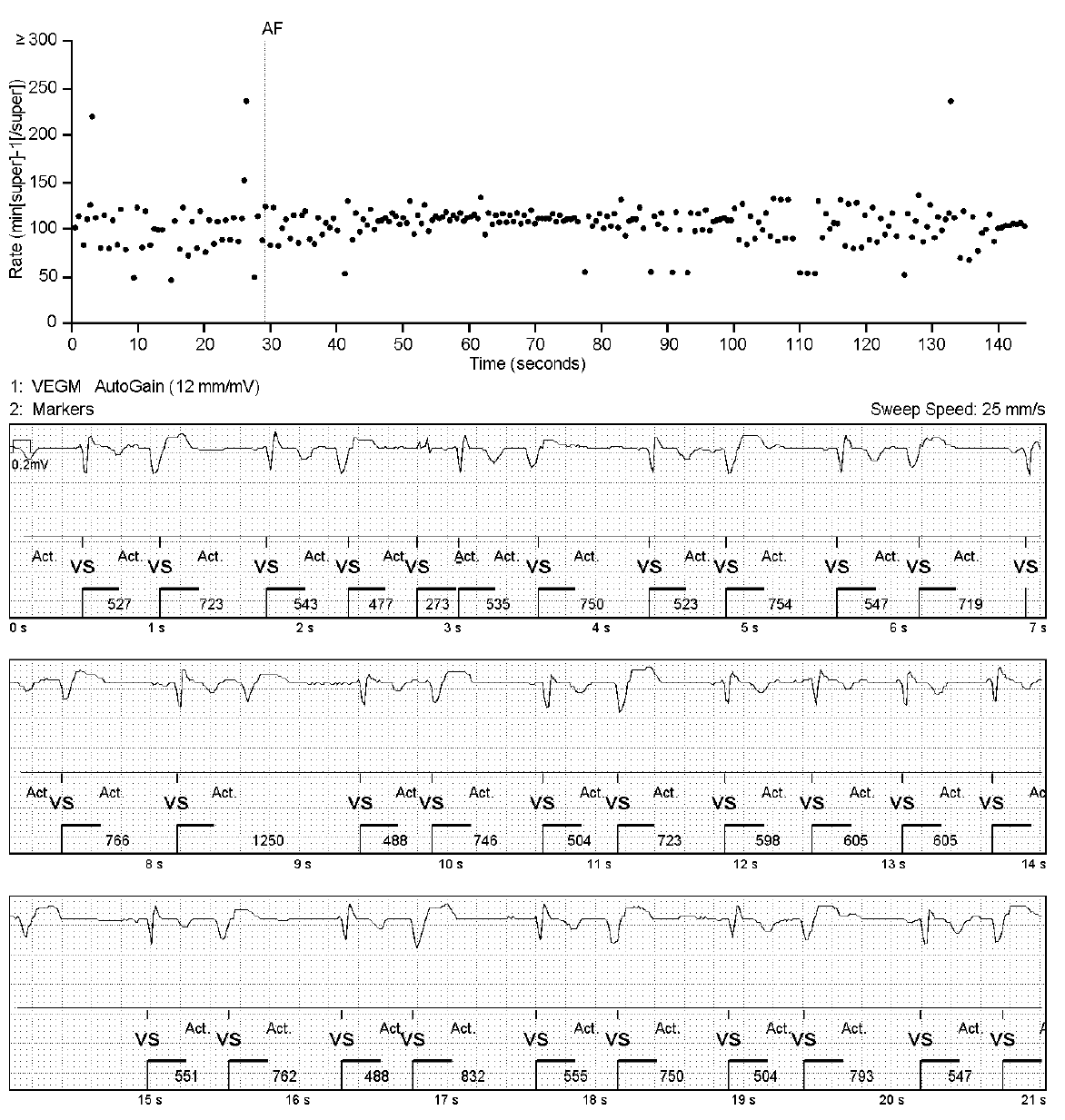


# **Figure S10.** Example of false positive episode due to ventricular ectopy.


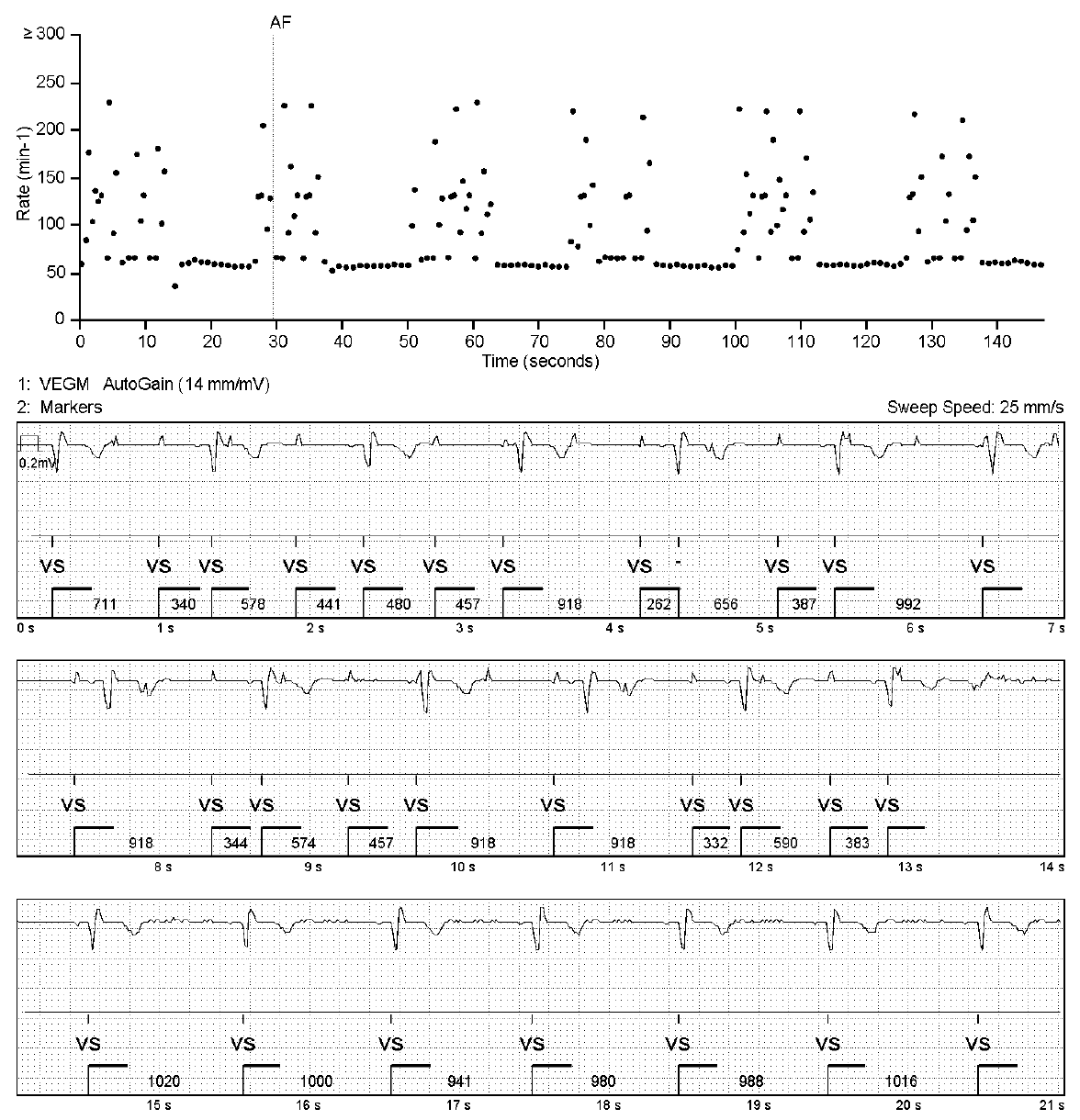


# **Figure S11.** Example of false-positive episode due to oversensing and ventricular ectopy.


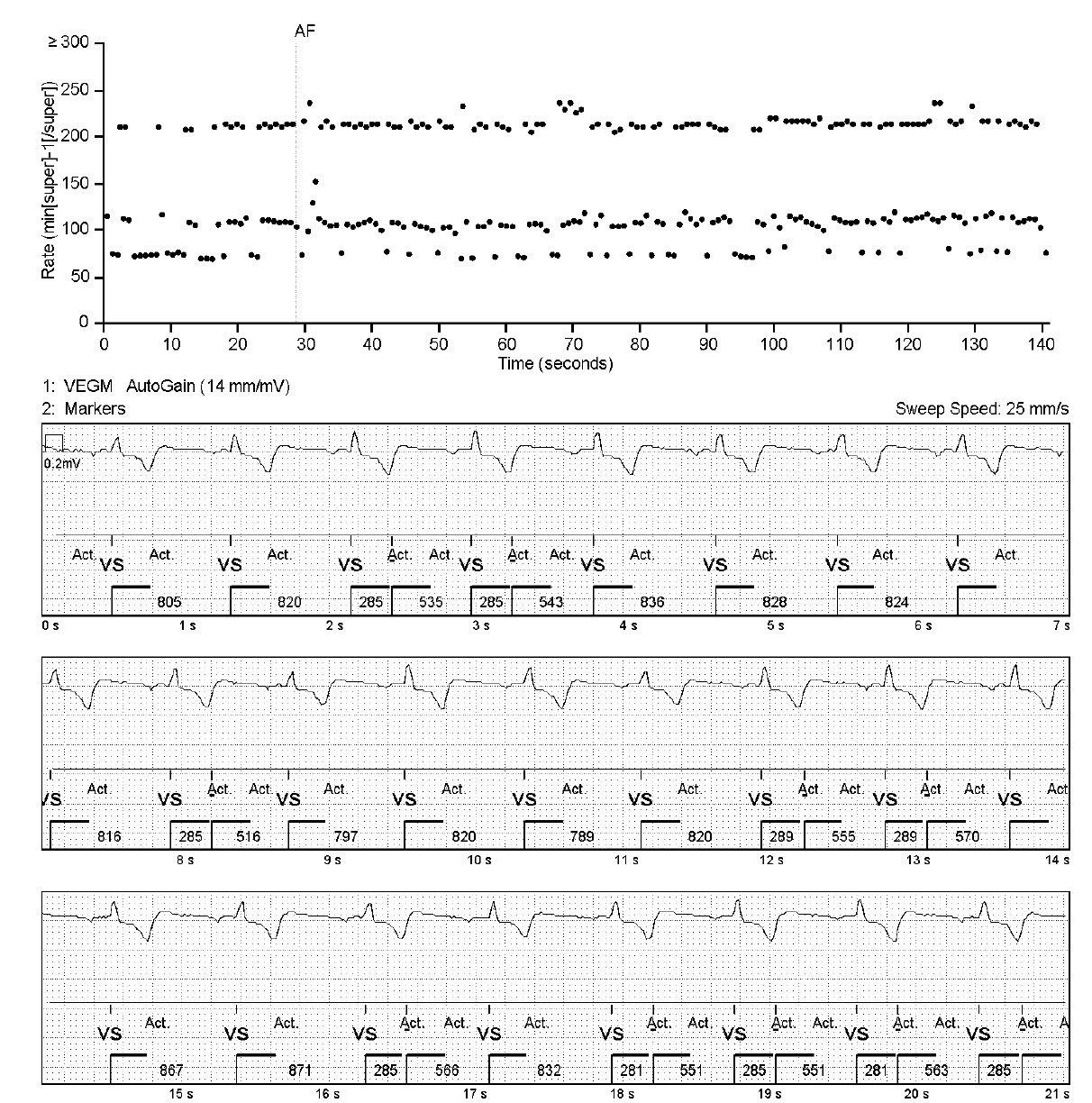


# **Figure S12**. Example of false-positive episode due to T-wave oversensing.


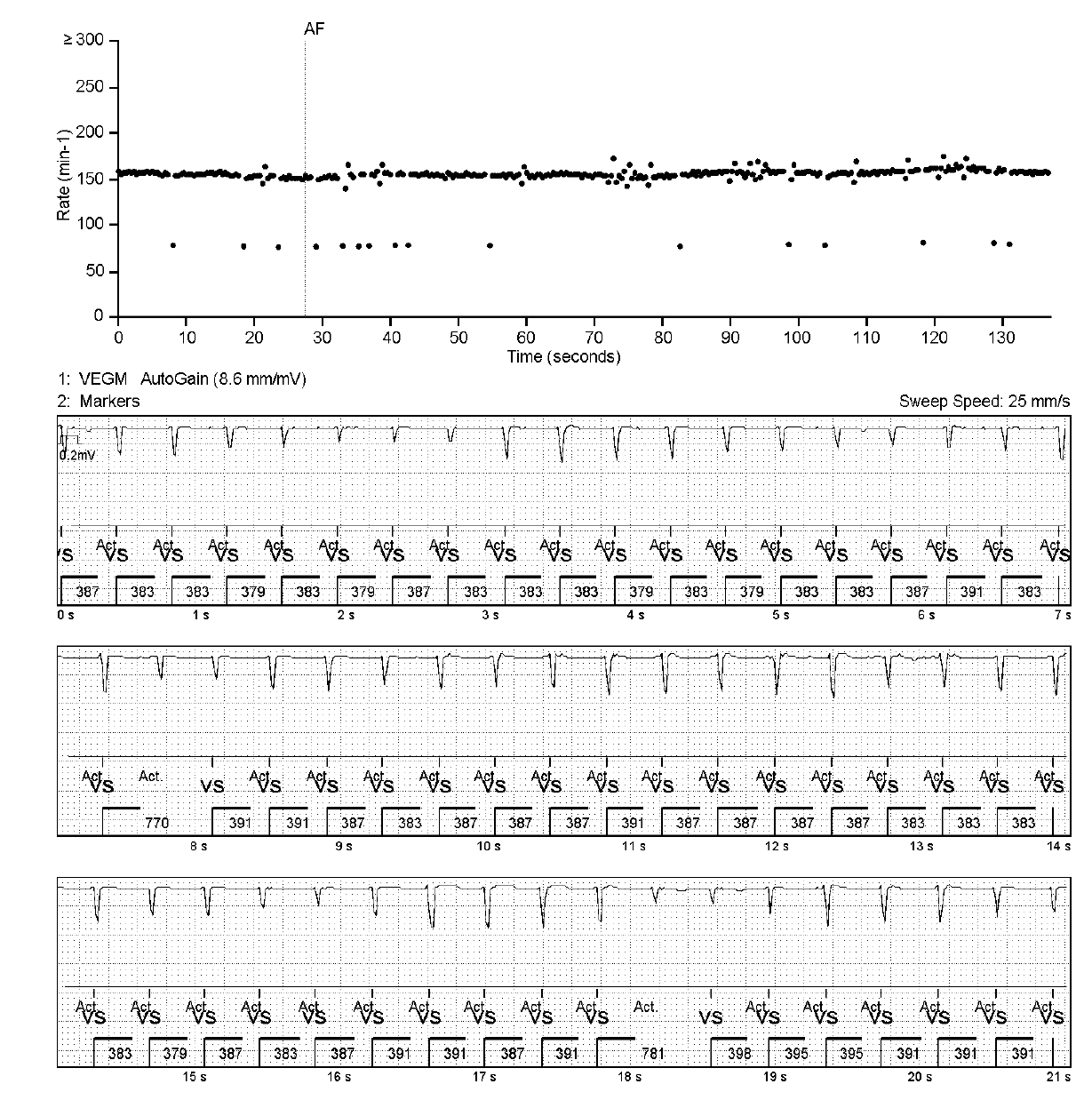


# **Figure S13** Example of false-positive episode due to undersensing.
